# Supplementary material for: Burden and risk factors of cutaneous leishmaniasis in a peri-urban settlement in Kenya, 2016
Source: PLoS One. 2020 Jan 23;15(1):e0227697. doi: 10.1371/journal.pone.0227697 (PMC6977748; doi:10.1371/journal.pone.0227697)
Supplement: S1 Checklist — (DOC) [file pone.0227697.s001.doc]

STROBE Statement— **Cases of Cutaneous Leishmaniasis in a peri-urban settlement in Kenya, 2016**

|  | | Item No | Recommendation |
| --- | --- | --- | --- |
| **Title and abstract** | | 1 | *(*a) Indicate the study’s design with a commonly used term in the title or the abstract  The abstract describes the study design as a “case control study” |
|  |  |  | (*b*) Provide in the abstract an informative and balanced summary of what was done and what was found  The abstract describes the ‘methods and findings’ of the study |
| Introduction | | | |
| Background/rationale | | 2 | Explain the scientific background and rationale for the investigation being reported  The scientific background and rationale are described in the ‘Introduction’, paragraphs 1-3. |
| Objectives | | 3 | State specific objectives, including any prespecified hypotheses  The specific aims of the study are stated in the ‘Introduction’, paragraphs 4. |
| Methods | | | |
| Study design | | 4 | Present key elements of study design early in the paper  The study design is discussed in paragraphs 4 of the ‘Introduction’ and paragraphs  2, 3 and 4 of the ‘Methods’ under sub-headings ‘study design’, ‘review of records’, and ‘case control study’ |
| Setting | | 5 | Describe the setting, locations, and relevant dates, including periods of recruitment, exposure, follow-up, and data collection  The study setting, location and dates are described in paragraphs 1 and 3 of ‘Methods’ |
| Participants | | 6 | *(*a) Give the eligibility criteria, and the sources and methods of case ascertainment and control selection. Give the rationale for the choice of cases and controls  Selection of the participants is discussed in paragraph 3, 5, 7, 8 and 9 of the ‘Methods’ section |
|  |  |  | (*b*) For matched studies, give matching criteria and the number of controls per case  Matching is described in paragraph 2 under ‘Case and control recruitment’ of ‘Methods section |
| Variables | | 7 | Clearly define all outcomes, exposures, predictors, potential confounders, and effect modifiers. Give diagnostic criteria, if applicable  The main outcome is described in paragraph 2 under ‘Review of records’ sub-section in the ‘Methods’ section. Other variables are described in paragraph 1 under “Data collection and analysis’ sub section in the ‘Methods’ section |
| Data sources/ measurement | | 8* | For each variable of interest, give sources of data and details of methods of assessment (measurement). Describe comparability of assessment methods if there is more than one group  Measurement of outcomes are discussed in paragraph 2 in ‘Case and control recruitment’ sub section and paragraph 1 in “Data collection and analysis’ sub section of the ‘Methods’ section |
| Bias | | 9 | Describe any efforts to address potential sources of bias  Sources of bias are addressed through random selection of respondents and by matching of controls to cases (paragraph 2 and 3 in ‘Cases and controls recruitment’ subsection, ‘Methods’ section) |
| Study size | | 10 | Explain how the study size was arrived at  The sample size estimation is described in the ‘sample size’ sub section under ‘Methods’ section |
| Quantitative variables | | 11 | Explain how quantitative variables were handled in the analyses. If applicable, describe which groupings were chosen and why  Use of variables and analysis is discussed in paragraph 1 and 2 under the ‘Data collection and analysis’ subsection |
| Statistical methods | | 12 | *(*a) Describe all statistical methods, including those used to control for confounding  Statistical analysis is discussed in paragraph 1 and 2 under the ‘Data collection and analysis’ subsection |
|  |  |  | (*b*) Describe any methods used to examine subgroups and interactions  Sub group analysis is discussed in paragraph 2 under the ‘Data collection and analysis’ subsection. Risk factors were categorised into three groups in the analysis: individual, indoor and outdoor factors |
|  |  |  | (*c*) Explain how missing data were addressed  Missing observations/data are discussed in paragraph 2 of the ‘Case and control recruitment’ in the ‘Methods’ section and is shown in the study flowchart (Figure 2). No individual variables are missing for individuals included in the analysis. |
|  |  |  | (*d*) If applicable, explain how matching of cases and controls was addressed  Matching is discussed in paragraph 2 of the ‘Case and control recruitment’ in the ‘Methods’ section |
|  |  |  | (*e*) Describe any sensitivity analyses  Last paragraph of ‘Data collection and analysis’ subsection of ‘Methods’ section describes regression analysis that was used to develop the final study model |
| Results | | | |
| Participants | | 13* | (a) Report numbers of individuals at each stage of study—eg numbers potentially eligible, examined for eligibility, confirmed eligible, included in the study, completing follow-up, and analysed  Data collection completion rates are discussed in paragraph 1 of the ‘Review of records’ sub section of ‘Results’ Section. The number of participants at each level completing each phase of data collection is also shown in the recruitment flowchart (Fig. 2). |
|  |  |  | (b) Give reasons for non-participation at each stage  Reasons for non-participation are discussed in paragraph 2 of the ‘Case and control recruitment’ sub section and in paragraph 1 ‘Review of records’ sub section of ‘Results’ Section and is represented in the study flowchart (Figure 2) |
|  |  |  | (c) Consider use of a flow diagram  See study flowchart (Figure 2) |
| Descriptive data | | 14* | (a) Give characteristics of study participants (eg demographic, clinical, social) and information on exposures and potential confounders  Participant characteristics are presented in paragraphs 1 and 2 of ‘Review of records’ sub section of ‘Results’ Section, paragraph 1 and 2 of ‘Case control’ sub section of ‘Results’ Section and further summarised in tables 1 and 2 |
|  |  |  | (b) Indicate number of participants with missing data for each variable of interest  Missing data are represented in the study flowchart (Figure 2) and in paragraph 2 of the ‘Case and control recruitment’ in the ‘Methods’ section. No individual variables are missing for individuals included in the analysis. |
| Outcome data | | 15* | Report numbers in each exposure category, or summary measures of exposure  Both numbers and percentages/proportions are reported throughout the Results  Section |
| Main results | | 16 | *(*a) Give unadjusted estimates and, if applicable, confounder-adjusted estimates and their precision (eg, 95% confidence interval). Make clear which confounders were adjusted for and why they were included  Raw (unadjusted) and adjusted results are presented for all outcomes throughout ‘Results’ section |
|  |  |  | (*b*) Report category boundaries when continuous variables were categorized  Shown in table 1 and 2 |
|  |  |  | (*c*) If relevant, consider translating estimates of relative risk into absolute risk for a meaningful time period  Not applicable |
| Other analyses | 17 | Report other analyses done—eg analyses of subgroups and interactions, and sensitivity analyses  Sub-group analysis is described in paragraph 1-4 while interactions and sensitivity analyses are described in paragraph 5 of ‘Risk factor analysis’ subsection of ‘Results’ section and further presented in tables 3 and 4 | |
| Discussion | | | |
| Key results | 18 | Summarise key results with reference to study objectives  Key results are summarized in paragraph 1 and 2 of ‘Discussion’ section | |
| Limitations | 19 | Discuss limitations of the study, taking into account sources of potential bias or imprecision. Discuss both direction and magnitude of any potential bias  Limitations are discussed in the last paragraph of ‘Discussion’ section | |
| Interpretation | 20 | Give a cautious overall interpretation of results considering objectives, limitations, multiplicity of analyses, results from similar studies, and other relevant evidence  Described in first paragraph of ‘Conclusion and recommendation’ subsection of the ‘Discussion’ section | |
| Generalisability | 21 | Discuss the generalisability (external validity) of the study results  The representativeness of the sample is discussed in ‘Conclusion and recommendation’ subsection. | |
| Other information | | | |
| Funding | 22 | Give the source of funding and the role of the funders for the present study and, if applicable, for the original study on which the present article is based  In the metadata | |
