# Supplementary material for: Burden and risk factors of cutaneous leishmaniasis in a peri-urban settlement in Kenya, 2016
Source: PLoS One. 2020 Jan 23;15(1):e0227697. doi: 10.1371/journal.pone.0227697 (PMC6977748; doi:10.1371/journal.pone.0227697)
Supplement: S1 Questionnaire — (PDF) [file pone.0227697.s002.pdf]

Cutaneous Leishmaniasis Questionnaire

Consent for the study has been given ☐ Yes ☐ No

PART I: Identifying Information

Questionnaire No ________________

Date of interview (dd/mm/yyyy): _____ / _____ /______

Interviewer name (Initials) _____________ Contact/Tel ______________________

Case Status ☐ Case ☐ Control

PART II: Demographic Information

2.1. Patient Initials _____________________

2.2. Sex ☐ Male ☐ Female

2.3. Date of birth (dd/mm/yyyy) _____/_____ /______

2.4. Residential village _______________________

2.5. Ward _________________________________

2.6. Education level

☐ None/No Formal Education ☐ Some Primary

☐ Continuing Primary ☐ Complete Primary

☐ Continuing Secondary ☐ Complete Secondary

☐ Tertiary

2.7. Employment status

☐ Employed (Formal) ☐ Unemployed

2.8. Occupation

☐ Housewife ☐ Student/School

☐ Livestock farming ☐ Mixed Farming

☐ House help ☐ Herdsman

☐ Business ☐ Hunting

☐ Charcoal Burning ☐ Game Warden/Guide

- [ ] Crop Farming
- [ ] Bee Keeper
- [ ] Lumber jack
- [ ] Mining/Stone Mason
- [ ] Other Occupation (Specify) _______________

2.9. Religion:

- [ ] Muslim
- [ ] Christian
- [ ] Hindu
- [ ] African tradition
- [ ] Other Religion (Specify) ________________

2.10. Total number of persons who reside in the household *(Specify number)* _______________

2.11. Record the GPS Coordinates for the household: ......°........‘........“ ......°........‘........“

PART III: Illness History

3.1. Date of Onset of Illness (dd/mm/yyyy) _____/_____ /______

3.2. Which of the following symptoms did you have? *Tick all that apply*

- [ ] Skin Sore (Ulcer)
- [ ] Mouth/Nostril Sore (Ulcer)
- [ ] Papule/Nodule (Bump/Lump)
- [ ] Loss of Appetite
- [ ] Rash
- [ ] Pruritus
- [ ] Glandular Swellings
- [ ] Scarring on the skin
- [ ] Bruising/Sloughing
- [ ] Nasal stuffiness
- [ ] Abdominal enlargement
- [ ] Recurrent infections
- [ ] Body weakness
- [ ] Bleeding gums/Nose
- [ ] Skin infections
- [ ] Fever
- [ ] Weight loss
- [ ] Other symptoms(s), Specify ______________________

3.2.1. If ‘yes’ to ‘skin sore’ above, specify location

- [ ] Head
- [ ] Neck
- [ ] Chest
- [ ] Abdomen
- [ ] Upper Limb
- [ ] Lower limbs
- [ ] Other body part (specify) _________________

3.3. Have you taken any medication for the complaints identified above?

- [ ] Yes
- [ ] No

3.4. If yes above, which ones?

- [ ] Antibiotics
- [ ] Painkillers
- [ ] Skin Ointment
- [ ] Traditional/Herbal medicine
- [ ] Dewormers
- [ ] Don't know
- [ ] Other medication (Specify) ____________

3.5. Have you sought care at a health facility for this illness?

- [ ] Yes
- [ ] No

3.6. If yes in 3.5. above, when did you first report to a health facility for this illness?

mm/yyyy _____ /______

3.7. If yes in 3.5. above, which facility did you first report to?

- [ ] Community Clinic
- [ ] Local Pharmacy
- [ ] Dispensary/Health Center
- [ ] Hospital
- [ ] Other facility (Specify) ____________

3.8. If 'No' in 3.5. Above, state the reason?

- [ ] Illness not serious
- [ ] Health facility too far
- [ ] Treatment Costly
- [ ] Hospital treatment ineffective
- [ ] Prefer alternative treatment
- [ ] Other reason (Specify) ____

3.9. Have you ever been diagnosed to have the following illnesses? Tick all that apply

- [ ] High blood pressure
- [ ] Diabetes
- [ ] Cancer
- [ ] HIV
- [ ] TB
- [ ] Sickle cell disease
- [ ] Leprosy
- [ ] Fungal skin infection
- [ ] Other Condition(s) (Specify)____________________

3.10. Usually when you fall ill, where do you seek treatment?

- [ ] Don't seek treatment
- [ ] Family member

☐ Community Health Worker ☐ Traditional Healer

☐ Chemist ☐ nearby Dispensary/Health Center

☐ County/Sub County hospital

☐ Other treatment (Specify) ________________________________

PART IV: Risk Factors Information

4.1. Did you travel away from usual residence in the last 12 months before this illness?

☐ Yes ☐ No

4.1.1. If yes, state the name of the county ____________________

4.2. Is there/Are there mosquito nets in your household that can be used while sleeping?

☐ Yes ☐ No

4.2.1. If yes, how many nets does your household have? (*Specify absolute number)* _____

4.2.2. How long ago did the household obtain the newest net?

☐ < 3 years ago ☐ > 3 years ago ☐ Can't tell

4.2.3. How frequently were you sleeping under an insecticide treated net at night during the last 3 months?

☐ Every night ☐ Some nights ☐ Rarely

☐ Never ☐ Don't know

4.2.4. Did you sleep under a mosquito net last night?

☐ Yes ☐ No

4.2.5. What is the type/brand of mosquito net did you sleep under last night?

☐ Long Lasting Treated Net *(Olyset, Parmanet, Supanet Extra etc)*

☐ Conventional net *(Kinga Net, Supanet, Rural Net, Mtumba net, Unbranded)*

☐ Can't tell

4.2.6. Has your net been 'treated' with insecticide since you got it?

☐ Yes ☐ No ☐ Can't tell

4.2.7. If yes, how long ago was the net treated?

☐ < 2 years ago ☐ > 2 years ago ☐ Can't tell

4.3. How frequently were you using insect repellent at night during the past 3 months?

☐ Every night ☐ Some nights ☐ Rarely

☐ Never ☐ Don't know

4.4. How frequently were you using fumigant at night during the past 3 months?

☐ Every night ☐ Some nights ☐ Rarely

☐ Never ☐ Don't know

4.5. At any time in the past 12 months, has anyone come into your dwelling/residence to spray the inside walls against insects?

☐ Yes ☐ No ☐ Don't Know

4.5.1. If yes, how long ago? mm/yyyy______/________

4.6. How often is the area around your home sprayed to control insects?

☐ Frequently/Monthly ☐ Once in 3-6 months

☐ Annually ☐ Never

4.7. Did anyone that you know have a similar illness, ulcers or bruises in the one year before your illness?

☐ Yes ☐ No ☐ Don't Know

4.7.1. If yes above, specify *(Tick all that apply)*

☐ Household member ☐ immediate neighbor (within 150 meters)

☐ Distant neighbor (outside 150 meters)

☐ Other person (Specify) ____

4.8. In a typical day, do you spend time outside your residence/house?

☐ Yes ☐ No

4.8.1. If yes, during which hours of the day? *(Tick all that apply)*

☐ Morning ☐ Afternoon ☐ Evening

☐ After sunset or at night

4.8.2. If 'Yes', specify where you spend most time. *(Tick all that apply)*

☐ In the home compound ☐ In the farm/garden

☐ In the forest ☐ In the market

☐ In the church/office/any other building

☐ Other place (Specify) ____

4.9. Do you have open containers with water holding capacity within the compound of your homestead?

☐ Yes ☐ No

4.10. Do you stay close to or come in contact with the following?

4.10.1. Domestic dog	☐ Yes	☐ No
4.10.2. Wild Jackals	☐ Yes	☐ No
4.10.3. Sheep	☐ Yes	☐ No
4.10.4. Goats	☐ Yes	☐ No
4.10.5. Rabbits	☐ Yes	☐ No
4.10.6. Cattle	☐ Yes	☐ No
4.10.3. Rock Hyraxes	☐ Yes	☐ No
4.10.4. Porcupine	☐ Yes	☐ No
4.10.5. Mongoose	☐ Yes	☐ No
4.10.6. Any Rodents	☐ Yes	☐ No
4.10.7. Anthill	☐ Yes	☐ No

4.10.7. Describe the nature of contact with these animals. *(Tick all that apply)*

☐ Herding ☐ Petting ☐ within the compound

☐ Hunting ☐ Park Visits ☐ Slaughtering/feeding

☐ Other contact (Specify) ______________________

4.11. Do you visit any nearby forest/thicket?

☐ Yes ☐ No

4.11.1. If yes above, describe the nature of your visit (Tick all that apply)

☐ Farming ☐ Hunting ☐ Nature walk/Tourism

☐ Charcoal burning ☐ Camping ☐ Fetching water/firewood

☐ Honey harvesting

☐ Other activity (Specify) ____

PART V: Environmental Observations

The enumerator to observe and record the following;

5.1. Roof/Ceiling type

- ☐ Grass/leaves thatch ☐ Mud
- ☐ Corrugated Iron ☐ Wood
- ☐ Other roof type (specify) ____

5.2. Nature of Floor

- ☐ Smooth plaster ☐ Cracked with holes and crevices
- ☐ Wooden ☐ Earthen
- ☐ Other floor type (Specify) _________________

5.3. Nature of the walls

- ☐ Smooth surface ☐ Cracked with holes and crevices
- ☐ Wooden ☐ Corrugated Sheet
- ☐ Earthern ☐ Other wall type (Specify) ____________________

5.4. State if the following are found **within 150 meters** from the residence:

5.4.1. Animal park/Game reserve?	☐ Yes	☐ No
5.4.2. Crop farm/Cultivated area?	☐ Yes	☐ No
5.4.3. Garbage pit/Open waste pit?	☐ Yes	☐ No
5.4.4. Animal/Farm manure?	☐ Yes	☐ No
5.4.5. Nearby river/open water source?	☐ Yes	☐ No
5.4.6. Nearby forest/Thicket?	☐ Yes	☐ No
5.4.7. Screens/Mesh on windows and doors	☐ Yes	☐ Partial/No

PART VI: Knowledge, attitudes and perceptions about Cutaneous Leishmaniasis

6.1. Have you heard about 'Cutaneous Leishmaniasis'?

- ☐ Yes ☐ No

6.1.1. If yes above, what was the source of your information? *Tick all that Apply*

- ☐ Health worker ☐ Media
- ☐ Community health volunteer ☐ A community member
- ☐ Other source (Specify) ___________________________________

6.2. How confident are you that you can hang a mosquito net in your household?

☐ Extremely confident ☐ Very confident

☐ A little confident ☐ Not at all confident

6.3. How important do you think it is for people to sleep under a treated net?

☐ Extremely important ☐ Very important

☐ A little important ☐ Not at all important

6.4. What is your opinion about the following statements?

6.4.1. People with ulcerating disease should be taken to a health facility for testing and treatment

☐ strongly agree ☐ somewhat agree

☐ somewhat disagree ☐ strongly disagree

6.4.2. Treated nets are safe to sleep under

☐ strongly agree ☐ somewhat agree

☐ somewhat disagree ☐ strongly disagree

6.4.3. People are at risk of insect bites only at night

☐ strongly agree ☐ somewhat agree

☐ somewhat disagree ☐ strongly disagree

(Thank the respondent)
