# Supplementary material for: Burden and risk factors of cutaneous leishmaniasis in a peri-urban settlement in Kenya, 2016
Source: PLoS One. 2020 Jan 23;15(1):e0227697. doi: 10.1371/journal.pone.0227697 (PMC6977748; doi:10.1371/journal.pone.0227697)
Supplement: S1 Leaflet — (PDF) [file pone.0227697.s003.pdf]

Cutaneous Leishmaniasis study information

Between January and February 2016, epidemiologists from DDSR and Field Epidemiology and Laboratory Training Program (FELTP) will be visiting health facilities and community units in Gilgil sub-county to investigate a suspected outbreak of Cutaneous Leishmaniasis. Cutaneous Leishmaniasis is a neglected tropical disease that is caused by the bite of a *Phlebotomus sandfly* and can cause a chronic and debilitating skin lesions in affected persons. The outbreak investigation follows reports of residents of Gilgil who have been falling ill with an ulcerative skin disease suspected to be cutaneous leishmaniasis. During this investigation, staff from National Ministry of Health (MOH) and Gilgil Sub county hospital will visit villages and health facilities in Morendati and Eburru/Mbaruk wards to;

1. Look for residents who have typical skin lesions (below figures)
2. Review hospital records to look for patients who have been treated for skin lesions in the past
3. Speak to identified patients with the skin lesions to collect demographic, health and various environmental exposures around their homes
4. Visit the homes of the suspected patients with cutaneous leishmaniasis and speak to other family members and their neighbors

Residents living in the identified villages and those with ulcerative skin lesions are encouraged to visit the hospital. The investigating team will seek permission from you to be included in the study.

HAVE YOU SEEN ANY OF THESE NEAR YOUR RESIDENCE?

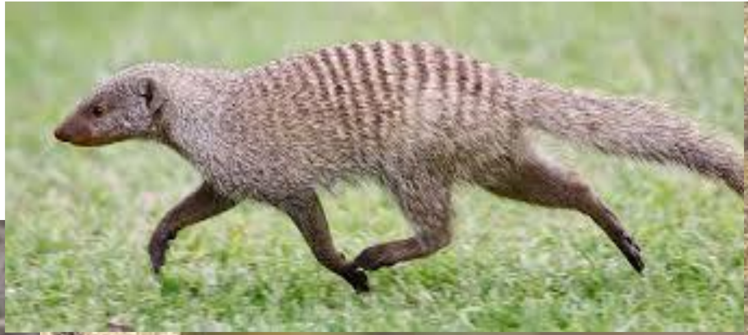

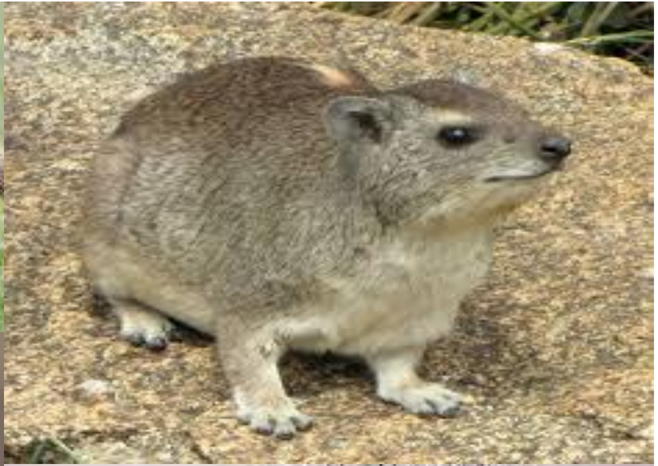

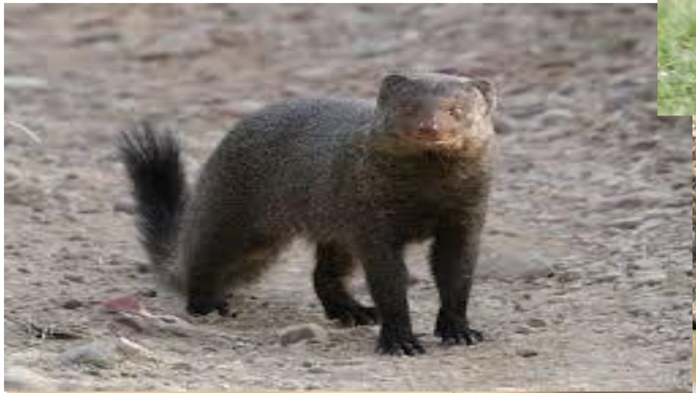

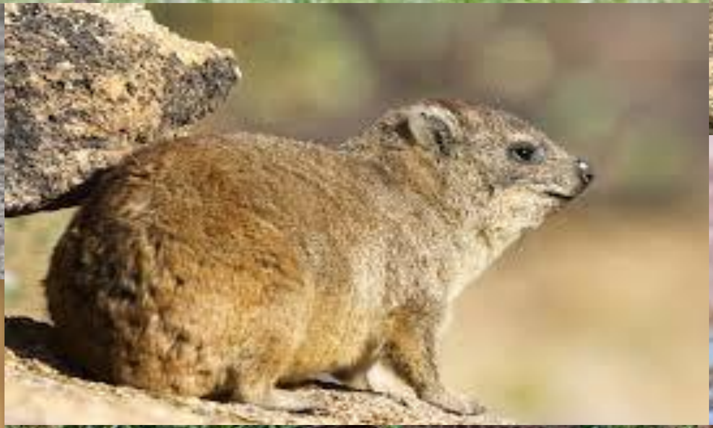

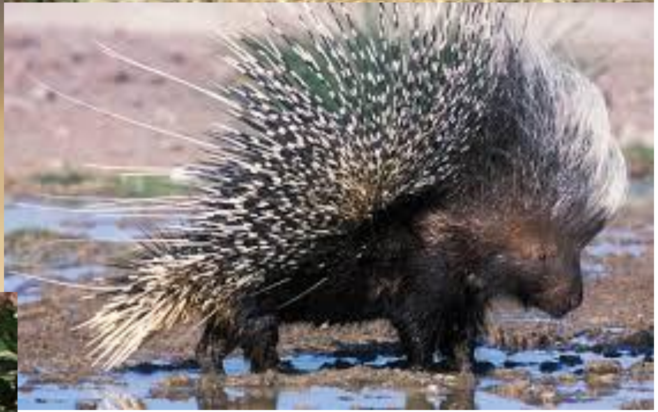

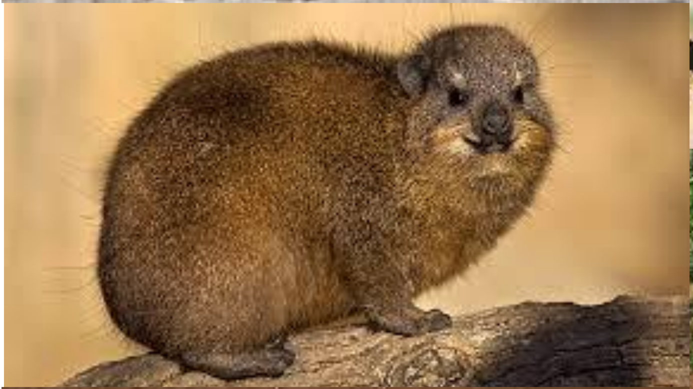

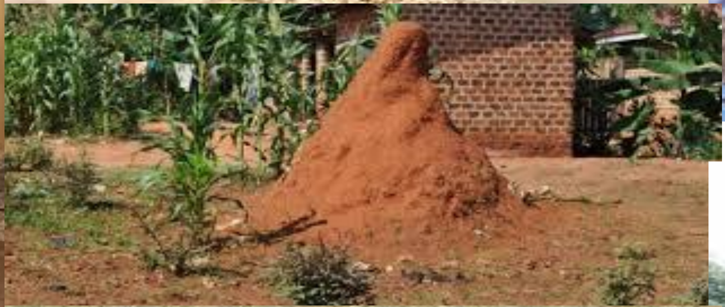

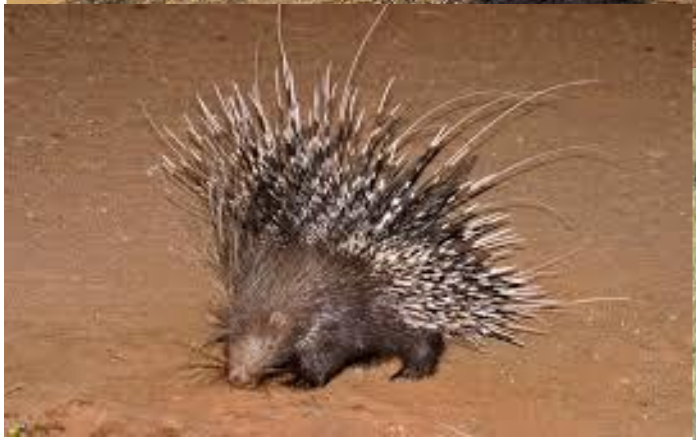

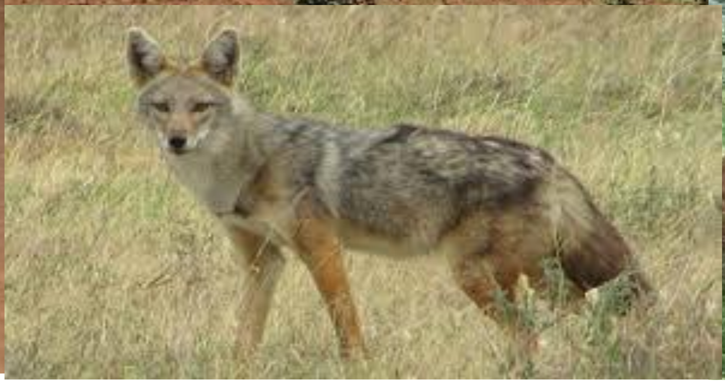

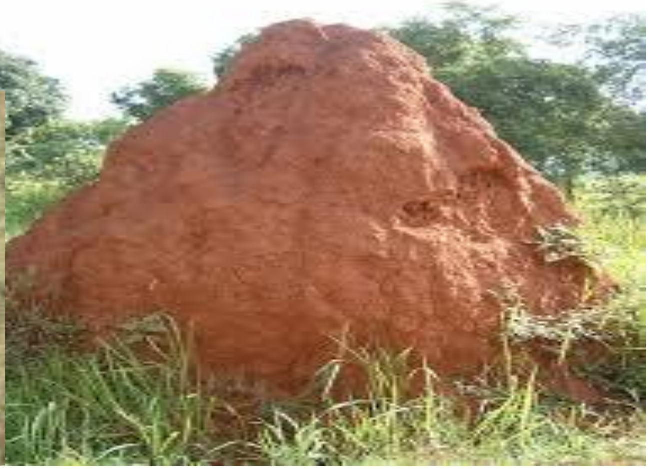

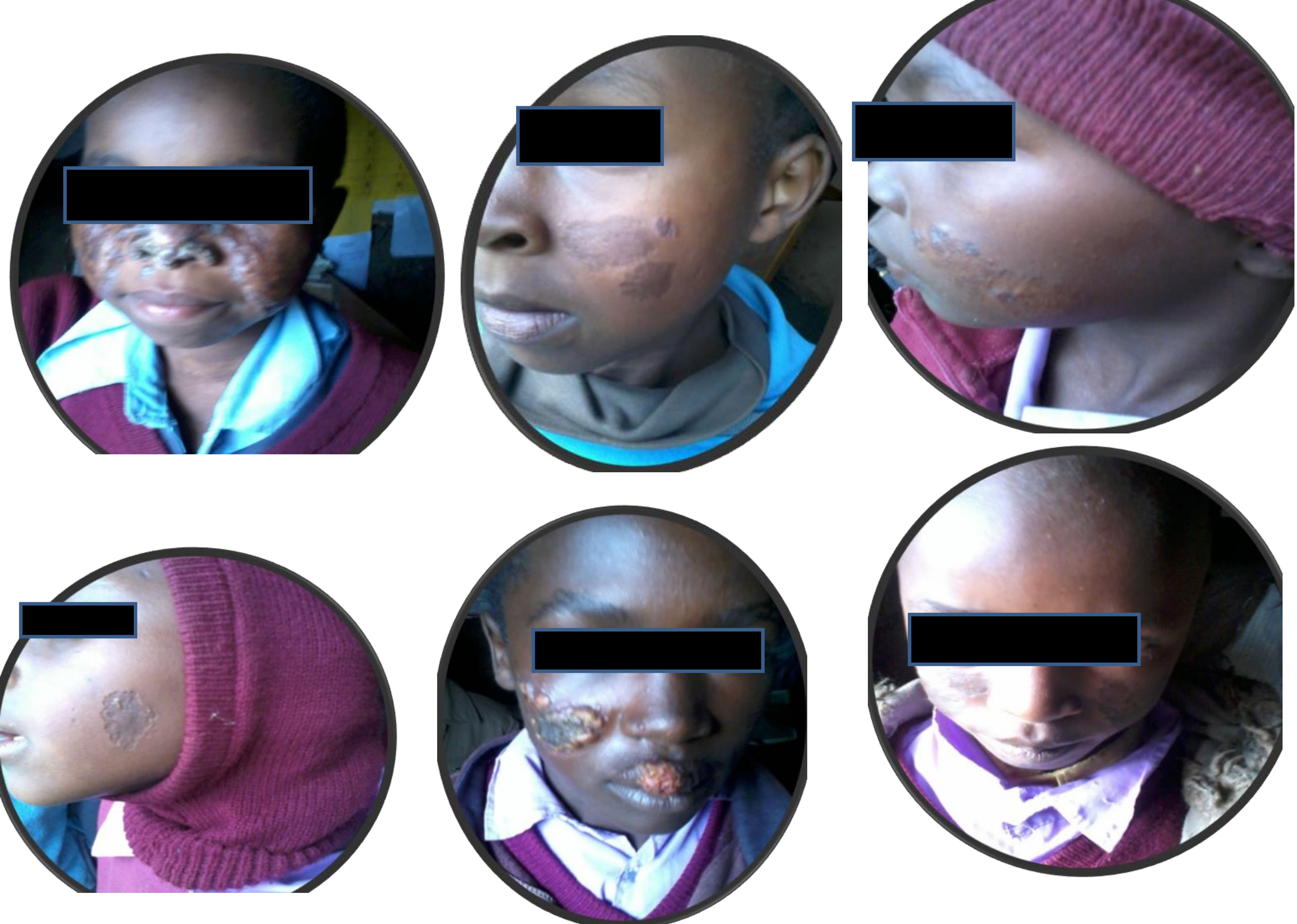

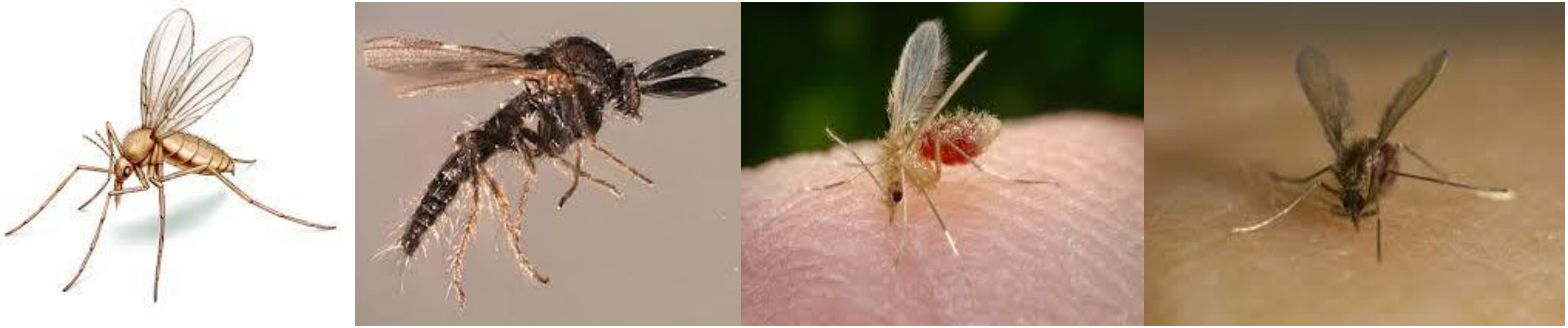

Sand Fly Vs Mosquito

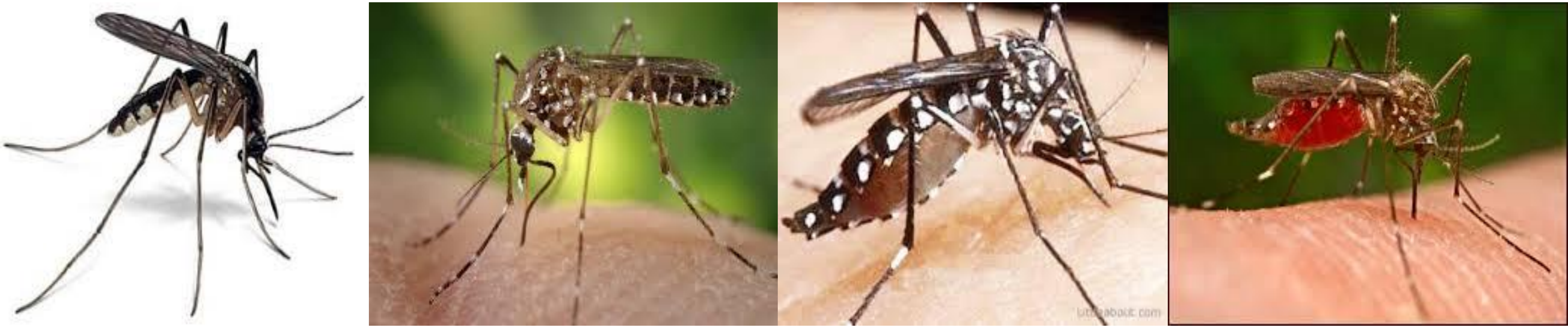

Sand fly vs. mosquitoes

- **Size-** smaller than the mosquitoes
- **Wings-** upright,lanceolate in shape and devoid of scales.
- **Legs-** longer than the body
- **Hairs-** Sandfly is a hairy insect
- **Hopping-** Generally hops and do not fly by choice
- If they flies, usually don't fly above 3 feet.
