# Supplementary material for: Burden and risk factors of cutaneous leishmaniasis in a peri-urban settlement in Kenya, 2016
Source: PLoS One. 2020 Jan 23;15(1):e0227697. doi: 10.1371/journal.pone.0227697 (PMC6977748; doi:10.1371/journal.pone.0227697)
Supplement: S1 Letter — (PDF) [file pone.0227697.s004.pdf]

MINISTRY OF HEALTH
DEPARTMENT OF PREVENTIVE AND PROMOTIVE HEALTH SERVICES
DIVISION OF DISEASE SURVEILLANCE AND RESPONSE

Telephone: Nairobi 2724951/ Fax 2724951
When replying please quote:

KENYATTA HOSPITAL GROUNDS
P. O. BOX 225 – 00202
KNH NAIROBI

Ref No: MOH/DPH/DSRU/REG/07/VOL. 1

21st January, 2016

To;
County Director of Health,
Nakuru County.

RE: Introductory Letter for MOH Field Investigation Team in Nakuru and Nyandarua County for suspected outbreak of Cutaneous Leishmaniasis

The Division of Disease Surveillance and Response (DDSR) of the Ministry of Health (MOH), received reports of suspected cases of Cutaneous Leishmaniasis in Gilgil sub-county and parts of Nyandarua County. As at early January 2016, a total of 53 suspected cases of Cutaneous Leishmaniasis were line listed. Among issues arising from the report are lack of knowledge on the extent of outbreak and challenges in diagnosis and case management of Cutaneous leishmaniasis. Following this report, MOH dispatched a team of Field Epidemiologists from DDSR and Field epidemiology and Laboratory Training Program (FELTP) to investigate the suspected outbreak of cutaneous Leishmaiasis in these counties

The planned Cutaneous Leishmaniasis outbreak investigation in Gilgil sub-county and parts of Nyandarua county will include the following activities

- Retrospective review of Hospital records for cases of Cutaneous Leishmania
- Active case search in the community to find out new suspected cases
- Health care worker Knowledge, Attitude and Practice (KAP) on cutaneous Leishmaniasis case management
- Community Knowledge, Attitude and Practice (KAP) on Cutaneous Leishmaniasis

The team intends to carry out the investigation for 10 days from 22nd January to 3rd February, 2016. This is therefore to kindly introduce to you the MOH team that will be investigating the suspected Leishmania outbreak in your county. The team will get in touch with the county health team ahead of travel for preparation of courtesy call and introductory meeting with the county and sub-county Health teams in due course

Thank you for your continued support

Yours sincerely,

For:

Dr. Ian Njeru
DISEASE SURVEILLANCE AND RESPONSE UNIT
