# Supplementary material for: Burden and risk factors of cutaneous leishmaniasis in a peri-urban settlement in Kenya, 2016
Source: PLoS One. 2020 Jan 23;15(1):e0227697. doi: 10.1371/journal.pone.0227697 (PMC6977748; doi:10.1371/journal.pone.0227697)
Supplement: Attachment — Submitted filename: Response to Reviewers_PLOSONE.doc [file pone.0227697.s007.doc]

December 3rd, 2019

The Academic Editor,

PloS ONE

Dear Abdalla M. Sammy,

**Re: Manuscript Re-submission**

We thank the reviewers for excellent comments on our manuscript entitled “**Burden and risk factors of Cutaneous Leishmaniasis in a peri-urban settlement in Kenya, 2016”.** We have addressed the reviewers’ comments and we are resubmitting the paper for consideration by the journal.

Below are detailed responses to the specific reviewers ‘comments

**REVIEWER #1.**

1. **Abstract**

“Of the 255 suspected cases of cutaneous leishmaniasis identified, females constituted 56% 28 (142/255) and the median age was 7 years (IQR 7-21). Cases were clustered in two locations: Gitare (28.6%, 73/255) and Kambi-Turkana (14%, 36/255) and a continuous transmission 30 pattern throughout the period was depicted”

The authors mentioned 255 cases. Then, they mention 73 case in Gitare and 36 case in Kambi giving a total of 109 case! This is really confusing!

**Response:** This statement has been revised to read “Cases occurred in clusters with up to 43% of cases originating from Gitare (73/255) and Kambi-Turkana (36/255) villages and a continuous transmission pattern throughout the period was depicted”

2. **Methodology**

Sample recruiting is very confusing and I was not satisfied with the explanation given to my previous concerns. In line number 141, the authors mentioned 172 cases. This number (172) was never mentioned again!

They recruited 41 cases from previous medical records assuming that those cases are CL. The medical record did not mention the diagnosis clearly and authors inferred from the description of lesions that those patients had CL. If the medical personnel who treated the patients when they visited the hospital could make the diagnosis, how authors could do so! Recruiting samples is very weak and hence the conclusions cannot be reliable, unfortunately.

**Response**: The section outlining recruitment of cases and controls and sample size estimation has been re-organized for clarity and flow. The sample size was determined by the number of eligible cases present in the study area during the study period. A sample size of 58 patients and 116 controls (2 controls per case) was therefore adopted to give the study at least 80% power at the 5% significance level and able to detect an odds ratio (OR) of 0.3 for an exposure present in 31% of controls. All the 41 cases that were selected from the outbreak line-list were first ascertained to be probable CL on the basis of typical skin lesions (a skin ulcer or a plaque) by experienced medical officers who were part of the study team.

**REVIEWER #3.**

1. Thank you for the revised revision, however, other comment need to be addressed

In the references section.

a. In the line 477, the reference number should be corrected to be 10 rather than 11
b. In the line 501, the reference number should be corrected to be 19 rather than 15
c. In the line 508, the reference number should be corrected to be 22 rather than 18

**Response**: The numbering has been corrected in the reference section of the revised manuscript

**REVIEWER #3.**

The manuscript has improved significantly and the link between the hospital based study and case control study is clearer now. The limitations have also been made more clear. There are still a few things I would like to point out

1. The CL abbreviation is still not done correctly (e.g. line 64, line 70, line 216)
2. Be consistent in formatting e.g. rift valley (92), Rift Valley (85), Sub-county (98) or sub county (100)
3. Typo's : line 98 (the study 2x), line 443-444 ("would be in theory be effective but would not be practical, (102) larva should be 'lava'

**Response**: These typos have been corrected

4. The statement on line 68-69 is from WHO I think (not from the review so change reference) and is also incorrect, an estimated 2 million new infections with leishmania (including 0.5 mil for VL) occur yearly, of which 1.5 million are for CL

**Response**: This reference has been corrected

5. The sample size is still not clear to me based on the new version of the manuscript and the reply to the comments. It seems all cases that could be found were included, and then a power calculation was done afterwards, rather then a sample size calculation beforehand, which would have guided when to stop recruiting patients. If this is the case I would phrase it more like below for clarity

"Based on the 58 patients and 116 controls (2 controls per case) , the study had 80% power to detect an odds ratio of 0.3 (using the 5% significance level for mosquito nets which was estimated to be present in 31% of controls."

**Response:** This section has been re-written for clarity and flow. The sample size was determined by the numbers of eligible cases present in the study area during the study period. A sample size of 58 patients and 116 controls (2 controls per case) was adopted to give the study at least 80% power at the 5% significance level and able to detect an odds ratio (OR) of 0.3 for an exposure present in 31% of controls. The exposure chosen was use of mosquito nets.

All other comments and typographical errors have been corrected. We hope you will now find the manuscript acceptable for publication in PLoS ONE.

Sincerely,

Isaac Ngere

E-mail: [isaac.ngere@wsu.edu](mailto:isaac.ngere@wsu.edu)

[ngereisaac@gmail.com](mailto:ngereisaac@gmail.com)

0724 963660
