# Supplementary material for: Burden and risk factors of cutaneous leishmaniasis in a peri-urban settlement in Kenya, 2016
Source: PLoS One. 2020 Jan 23;15(1):e0227697. doi: 10.1371/journal.pone.0227697 (PMC6977748; doi:10.1371/journal.pone.0227697)
Supplement: Attachment — Submitted filename: Response to Reviewers_PLOSONE.doc [file pone.0227697.s008.doc]

December 16th, 2019

The Academic Editor,

PloS ONE

Dear Abdalla M. Sammy,

**Re: Manuscript Re-submission**

We have addressed the editor’s comments on our manuscript entitled “**Burden and risk factors of Cutaneous Leishmaniasis in a peri-urban settlement in Kenya, 2016”** and we are resubmitting the paper for consideration by the journal.

Below are responses to the specific comments from the editor;

**EDITOR’S COMMENTS**

- The authors should provide the figures in 300 dpi as per Journal regulations; all figures looks unsuitable as it currently stands.

This has been addressed. The figures have been edited to the right format and uploaded via the PACE digital diagnostic tool and checked for compliance to journal requirements

- Table 3: :"Net use by the individual", do you mean the bednet?

This table has been edited to read ‘individual using bed net’. Other variable names in the table have been revised accordingly

- Please carefully revise the language of your manuscript in all sections and tables.

We have revised the language and flow of the entire manuscript, including the tables.

All other comments and typographical errors have also been corrected. We hope you will now find the manuscript acceptable for publication in PLoS ONE.

Sincerely,

Isaac Ngere

E-mail: [isaac.ngere@wsu.edu](mailto:isaac.ngere@wsu.edu)

[ngereisaac@gmail.com](mailto:ngereisaac@gmail.com)

0724 963660
